# Technology assessment and resource allocation for predictive genetic testing: A study of the perspectives of Canadian genetic health care providers

**DOI:** 10.1186/1472-6939-10-6

**Published:** 2009-06-18

**Authors:** Alethea Adair, Robyn Hyde-Lay, Edna Einsiedel, Timothy Caulfield

**Affiliations:** 1 Health Law Institute, University of Alberta, Edmonton, Alberta, Canada; 2 Faculty of Communications and Culture, University of Calgary, Calgary, Alberta, Canada

## Abstract

**Background:**

With a growing number of genetic tests becoming available to the health and consumer markets, genetic health care providers in Canada are faced with the challenge of developing robust decision rules or guidelines to allocate a finite number of public resources. The objective of this study was to gain Canadian genetic health providers' perspectives on factors and criteria that influence and shape resource allocation decisions for publically funded predictive genetic testing in Canada.

**Methods:**

The authors conducted semi-structured interviews with 16 senior lab directors and clinicians at publically funded Canadian predictive genetic testing facilities. Participants were drawn from British Columbia, Alberta, Manitoba, Ontario, Quebec and Nova Scotia. Given the community sampled was identified as being relatively small and challenging to access, purposive sampling coupled with snowball sampling methodologies were utilized.

**Results:**

Surveyed lab directors and clinicians indicated that predictive genetic tests were funded provincially by one of two predominant funding models, but they themselves played a significant role in how these funds were allocated for specific tests and services. They also rated and identified several factors that influenced allocation decisions and patients' decisions regarding testing. Lastly, participants provided recommendations regarding changes to existing allocation models and showed support for a national evaluation process for predictive testing.

**Conclusion:**

Our findings suggest that largely local and relatively ad hoc decision making processes are being made in relation to resource allocations for predictive genetic tests and that a more coordinated and, potentially, national approach to allocation decisions in this context may be appropriate.

## Background

Canada has invested significant funding for genetic research, yielding new technologies and, in turn, increased patient demand [1]. As genetic technologies continue to be introduced into mainstream health care, assessment raises difficult issues for health care providers and policymakers [2]. Broadly speaking, these parties are faced with allocating resources for an ever-expanding list of new genetic health technologies in the context of the existing and future pressures to the health care system. Assessment for genetic technologies raises questions with regard to legal and ethical duties of care owed by health care providers involved in the provision of genetic services [3-5]. It also faces the practical challenges of generating and incorporating robust evidence into health care resource allocations [6], finding the appropriate balance between quantitative and qualitative information [7], and setting uniform evaluation standards across highly variable testing and health outcomes [6]. A variety of new assessment models have developed alongside emerging genetic technologies [8-10]. However, with only preliminary policy development for resource allocation for such technologies [11-13], Canadian technology assessment has evolved as a predominantly informal, ad hoc process.

Health providers play a key role in decisions about the utilization of genetic technologies and, as such, their perspective on genetic medicine is of value [14,15]. For the purposes of this paper genetic medicine has been defined as "the study of genetic mechanisms including the genetic basis for human diseases, and the development of genetically based tests and therapies" [16].

In 2007, the authors conducted a study which consisted of a series of semi-structured interviews with senior lab directors and clinicians at publicly funded Canadian predictive genetic testing facilities. These interviews were designed to provide key information regarding the allocation of resources for predictive genetic testing and associated ethical, legal and social issues. More specifically, they explored existing provincial funding structures, criteria and factors that influence resource allocation decisions, relationships between primary health providers and government representatives, the role the media plays in influencing patients' requests for genetic tests, and the strengths and weaknesses of the current regulatory framework within which these facilities operate. Policy recommendations regarding changes to resource allocation models and the viability of a national evaluation process for predictive genetic tests were also examined. Information obtained from these interviews has been used to inform ongoing research investigating resource allocation of emerging genomic technologies, the end goal of which is to formulate a list of funding policy recommendations to assist in resource allocation decisions.

The facilities and individuals providing predictive genetic testing – or tests that help determine an individual's predisposition to a particular health condition or disease – is a relatively small community in Canada, and those interviewed were identified by their community as leaders in this context. The interviews specifically focused on predictive genetic testing for adult onset hereditary diseases, such as breast and colon cancer, and Huntington's disease.

## Methods

This paper presents the results of a study exploring senior lab directors' and clinicians' perspectives on resource allocation for predictive genetic testing in Canada and associated ethical, legal and social issues. As the community sampled was identified as being relatively small and challenging to access, purposive sampling coupled with snowball sampling methodologies were utilized [17,18].

A preliminary list of potential interview participants was created through consultation with project collaborators and senior lab directors, with reference to the Canadian College of Medical Geneticists membership directory. A total of 45 individuals were invited by email to gauge interest in completing the interviews, and were subsequently contacted by telephone. Of the original sample of 45, the authors were able to speak with 31 potential participants. During the initial discussions with these individuals, the authors asked them to identify the best member within their local genetic testing community to interview. Eleven individuals referred the authors to other genetic community members – all of whom were on the original contact list – three declined to participate, and scheduling conflicts prevented the participation of one, resulting in a final sample of 16. Between January-March 2007, semi-structured interviews were conducted with individuals from the final sample either by telephone (14) or by email (2) according to the respondent's preferences, with the average telephone interview lasting approximately 40 minutes. Participants were drawn from British Columbia, Alberta, Manitoba, Ontario, Quebec, and Nova Scotia, and the final sample was composed of eight lab directors and eight clinicians.

The semi-structured interview instrument was developed in consultation with project collaborators and senior clinicians. In total 13 questions were included, and data was collected through a series of both open and closed ended questions. All participants received a copy of the survey instrument in advance of their scheduled telephone interview and all interviews were conducted by the same individuals. They were tape-recorded and open ended responses were transcribed at the time the interviews were conducted. For those that participated via telephone, consent to participate was obtained before the survey commenced, and was recorded on tape. For those participants that completed the survey via email, consent to participate was explained to be implicit in their completion of the survey. Given the small sample size, results are descriptive and have no transferrable statistical significance. Open ended questions were transcribed and general themes were simply identified. For closed ended questions, responses were simply counted on a question by question basis and compared to the total sum of responses given for each corresponding question. Participants were also given the opportunity to discuss any additional topics or issues they wished to raise.

The University of Alberta Art, Science and Law Research Ethics Board approved the research protocol. Responses were de-identified by assigning provincial locations and a number to each participant. Information from the study that could render individuals identifiable in any way has not been included in published results.

## Results

### Policy at National, Provincial and Laboratory Levels

Funding for genetic testing varies widely across Canada [1]. The Canadian health care system is essentially comprised of a grouping of provincial systems and health care delivery is considered a provincial matter with each province allocating funds for services slightly differently. These structural variations help to create different approaches to the funding of genetic tests. Surveyed lab directors and clinicians were asked to explain how predictive genetic tests were funded at their facility. Their responses identified two predominant funding models. In provinces such as Quebec, Nova Scotia, Alberta and Ontario, participants explained that funding is allocated to hospital and regional budgets. The hospital or region in turn makes decisions about the overall yearly budget for their respective genetic laboratories. For example, one participant from Ontario explained:

What happens in Ontario, the genetic testing comes out of the hospital budget, the hospital gives X number of dollars to genetic testing. In other provinces the funding goes directly for genetic testing. In some cases, there is a mix. For BRCA1 there is targeted [funding] for a few centres. For the majority of the other 200 DNA tests it comes through the global hospital budget.

In provinces such as British Columbia and Manitoba, funding is allocated directly to the genetic laboratory under the province's respective Ministry of Health. The hospital does not intervene in resource allocation decisions. A participant from one of these provinces explained:

Predictive genetic tests are funded through the Molecular Diagnostic Laboratory budget which in turn is provided through the local regional health authority budget.

In all provinces surveyed, the lab directors or head clinicians generally played a significant role in allocating the funds they received to specific tests and services, influencing day-to-day utilization decisions.

It was also found that participants were largely making such everyday allocation decisions without consultation with government representatives. Respondents were queried on the nature of their relationship with national and provincial government representatives. Participants were asked: "Who in the federal and/or provincial government has a role in the implementation of predictive genetic tests and resource allocation at your facility? Do you work with them?" While, a third of respondents (5/16) were able to identify a process for contacting the provincial government, two thirds of participants (11/16) indicated, in fact, that there was no one in the federal or provincial government that they could pinpoint as having a role. For example, one person stated "the contact person changes all the time, right now no one actually knows who the contact person is." Another explained:

No one in the provincial government, as far as I know, has a role in implementing predictive genetic tests. We, geneticists, have written proposals to implement genetic tests either in-house or through an out-of-centre laboratory. I do know that only a handful of proposals are brought forward to the attention of the provincial Minister of Health's office. Which proposals that are moved forward is decided by senior regional health authority officials and which ones are ultimately funded is decided by governmental officials.

These responses support a picture of an allocation process that is largely regional and localized to the health care community.

Respondents from across Canada also revealed that allocation decisions related to the distribution of funds for predictive genetic tests at the laboratory level were being influenced by a variety of factors. Underfunding for genetic tests was a recurrent theme in the discussion. When asked "are there any tests that have received premature funding," only one response indicated such incidents (1/16). In contrast, when asked, "are there tests that are not currently funded that your facility would like to provide" almost all respondents (15/16) answered in the affirmative. Participants were also asked to rate on a scale of 1–5, with 5 being very important and 1 not important, a list of potential factors influencing resource allocation decisions for predictive genetic testing at their facility These included: cost effectiveness, length of wait times, access to the appropriate equipment, access to new technologies, evidential basis, availability of preventative strategies, ethical/legal consideration, and media coverage. Participant responses varied, but for each of the factors listed, over half of the participants gave a rating of 3 or higher, with evidential basis and cost effectiveness consistently receiving the highest ratings. Such ratings suggest that all the above factors influence resource allocation decisions for predictive genetic testing in Canada at the laboratory level.

A number of participants then offered recommendations for changes to resource allocations specifically related to the mechanisms used to fund predictive genetic tests. When asked: "How do you think predictive genetic tests should be funded?" participants expressed a range of views. Some participants (5/16) indicated a preference for funding on a per test basis, *e.g*., "We need to be funded for each test that we are doing." A small minority (2/16) identified a preference for specific set of funds allocated to each type of genetic test, *e.g*., "It would be better to have funds specifically allocated for [predictive genetic testing] so if there is a need for further funding the lab would have the ability to justify itself."

Others noted (5/16) that the current mechanism through which funds were being allocated was satisfactory, in particular those that were funded under a global budget. One of these participants supported the evaluation process at a hospital-based laboratory, whereby funding for genetic testing was allocated after an evidence-based committee review, explaining:

We have a committee that decides if it is a novel test. The person that is requesting this goes in front of a committee that decides if it is scientifically sound, and how it is going to impact on our hospital... This [is] fairly straightforward.

The remainder of those interviewed (4/16) explained their funding systems but did not comment on their level of satisfaction.

The study sought to discover whether there was support for further policy development for technology assessment for genomic health technologies, initiated at the national level. When asked: "Do you think a national evaluation process for all predictive testing would be useful?" three quarters of participants (12/16) responded in the affirmative, *e.g*., "Canadian national guidelines would be a good place to start" and " [it would be] useful to have national standards on what is paid for and for those to be informed by expertise." Of those interviewed, the difficulty of coordinating or implementing a national evaluation process was raised (6/16), including by four of the participants who were supportive of new standards, *e.g*.:

We live in a country where healthcare is the provincial jurisdiction. Whatever we say national is unlikely to impact resources. But the federal government could put money into the Canadian College of Medical Genetics to push standardization, to ensure tests are performed in an excellent fashion, standards for genetic counseling – that would be great.

These participants also emphasized the need for sensitivity to the local and provincial nature of health care, cautioning, *e.g*., " [there are] very big regional differences and pressures per region."

### Legal, Ethical and Social Issues

The study also focused on the relationship between resource allocation and the legal and ethical duties of care owed by health care providers involved in the provision of genetic services in the context of resource allocation policies [4,6,19]. Respondents were asked, "Assuming patient consent, who makes the decision about whether a predictive genetic test will be done?" Four options were provided: the primary care physician, the specialist, the genetic counselor or the laboratory. The majority of participants (11/16) indicated that more than one professional is involved in this process. Many respondents suggested that determining the professional responsible for making a decision on testing is dependant on the type of test. *e.g*., "There are certain tests where we would allow the primary care physician to make the decision, and there are certain tests where we need a genetic counselor to make the decision." One respondent deferred to policies rather than individual decision-making, suggesting "I am not sure if there is much of a decision because there are protocols in place so if they meet the protocols they get the test." This variability raises questions about the ethical responsibilities of each professional, as well as questions with regard to clarity regarding who has ultimate legal responsibility in relation to the decision making process.

Respondents were also questioned on a scenario that poses legal and ethical challenges for health care providers. They were asked "When should a patient's desire to have a specific test overrule the clinical indication for the test?" and offered three options – always, sometimes, never. Three quarters of respondents (12/16) replied that this decision should be made sometimes, depending on other social factors or the strength of the request. Generally, participant responses focused on the standard of care that they should be providing as health care professionals, *e.g*., "there are times when people are so stressed out that that might be an indication on its own." While those surveyed offered little insight into the nature of the legal obligations they owed in this role, the responses seem to represent a thoughtful balancing of an evidence based approach and a consideration of ethical obligations.

The study also investigated genetics health care providers' perceptions of social factors influencing patient decisions about predictive genetic testing. Respondents were asked to rate the influence of patients' concern about genetic discrimination, impact on family members, ability to obtain insurance, and additional costs associated with a positive result, on decisions regarding testing [20-22]. Providers offered a range of perceptions on patients' influences. In particular, genetic discrimination was viewed as both, *e.g*., "A high concern for most patients" and something that " [does not come] up all too often, in our health care system." Overall, half of the participants (8/16) rated the influence of patients' concern about genetic discrimination, as a 3 or higher, on a scale of 1 to 5, with 5 being very important and 1 not important. Interestingly, a couple respondents (2/16) also commented that factors such as genetic discrimination and the ability to obtain insurance are not normally influential until raised by health providers: "That's often an issue, again often one that we raise that they may not have thought about." In other words, providers expressed that patients were often unaware of the potential for genetic discrimination or difficulty obtaining insurance before their genetic health provider raised these issues.

Finally, the study queried genetic health care providers' perceptions of the influence of the media. When asked to rate the influence of "media portrayals of genetics on patient requests for genetic tests," on a scale, with 5 being very influential and 1 not influential, a majority of respondents (11/16) affirmed that media portrayals were either rated as a 4 or a 5. These findings emphasize the role of the media as an information conduit and a public education tool that assists in the introduction of new genetic technologies; one participant noted " [the media] have done a better job than the medical community." Respondents were also cautious about the tendency for information on genetic issues to become disproportionately emphasized, *e.g*., "The press is great for advocacy, not so good on issues requiring a perspective. That said, the media does keep us on our toes and does have a role." These findings confirm what other research has found regarding the media in the context of genetic technologies [23-25], including an awareness of genomics hype through media attention, and underscore the importance of accounting for social pressures on resource allocation structures.

## Discussion

Internationally, the reviews of appropriate standards for robust decision making processes and frameworks for the delivery of new health technologies, including genetic testing, are emerging. For example, in 2006 the New Zealand government examined the role of evidenced based medicine in health boards' decision making processes, considering the factors that influenced decisions by health boards; national and regional decision making processes; and views on evidence based medicine from the clinician's perspective [26]. This study was motivated by the need to develop further insight into Canadian resource allocation issues in the context of predictive genetic testing. The value of understanding the provider perspective and the impact it might have on allocation policy is key. Genetic professionals are a vital part of the allocation process, making daily clinical decisions regarding utilization and playing a unique role in policy development.

Despite some controversy about the clinical utility of genetic testing [27,28] and the robustness of the technology assessment surrounding their implementation [9,28], the perception from Canadian clinicians is that genetic tests are not receiving premature public funding and that, in fact, more tests should receive funding. From the health care professional perspective, the pressure will be for more funds and increased testing. Legal and ethical factors may also drive utilization. These pressures would likely be most effectively balanced by the development of independent, evidence based allocation policies and clinical practice guidelines.

This study has a number of limitations. In addition to a small sample size, and therefore limited generalizablity of results, telephone and email surveys have the potential to yield different results as they are each distinct modes of data collection. Despite these limitations the findings provide baseline data on key players' perspectives on factors and criteria that influence and shape the allocation of resources for publically funded predictive genetic testing in Canada.

## Conclusion

As new genetic tests continue to become available to the health care system, the importance of an effective decision making process for distributing the limited quantity of available health care funding will remain at the forefront. While it is important to recognize the value of the regional and provincial decision making that is currently being undertaken with regard to allocating funds for new technologies in Canada, the role of national policies and guidance should also be considered. In the United Kingdom, the evaluation of and decision to allocate resources to new genetic tests is completed under the umbrella of the UK Genetic Testing Network (UKGTN) [29], a national framework for the delivery of genetic tests. Under this system, when a laboratory or health care client wants a new genetic test to be adopted by the UKGTN, a "gene dossier" must be submitted which includes information on the "disease prevalence, test characteristics, utility and validity of the test" and which is reviewed by the Steering Committee that prioritises the requests [29,30].

This unified approach stands in contrast to the largely local and relatively ad hoc decision making processes described by genetic providers interviewed in this study. As discussed above, few of those interviewed – despite being leaders within the clinical genetics community – could identify any relevant provincial or federal government contact. In addition, while a broad range of often conflicting factors impact allocation decisions, including for example, media coverage, patient demands, and legal and ethical issues, there is no single strategy for prioritizing needs or allocating funds. It is not surprising, then, that there was general agreement concerning the value of national policies. Indeed, our data lends support to the development of a more coordinated and, potentially, national approach to allocation decisions in this context. That is not to say that participants favored overriding current processes and the multiple variants at play in stakeholder decisions. Participants acknowledged the complexity of coordinating a national review in Canada given the provincial jurisdiction of health care funding. With this in mind, however, a national forum would provide the opportunity to support these decision making processes while clarifying prioritization criteria, encouraging provincial interrelationships, and promoting standardization.

## Competing interests

The authors declare that they have no competing interests.

## Authors' contributions

TC and RHL conceived of and designed the study. AA and RHL conducted data collection and contributed to the writing of the paper. EE assisted with data analysis. TC supervised data collection and analysis. All authors read and approved the final manuscript.

## Pre-publication history

The pre-publication history for this paper can be accessed here:



## References

[B1] Silversides A (2007). The wide gap between genetic research and clinical needs. MAJ.

[B2] Haga S, Willard H (2006). Defining the spectrum of genome policy. Nat Rev Genet.

[B3] Chenier J (2006). Resource allocation and the standard of care of physicians. Can Bar Rev.

[B4] Burke W, Press N (2006). Genetics as a tool to improve cancer outcomes: Ethics and policy. Nat Rev Cancer.

[B5] Potter B Approaches to addressing ethical, legal, and social issues in health technology assessment: Application to prenatal and newborn screening. Health Technology Assessment International 4th Annual Meeting, 17–20 June 2007, Barcelona, Spain (refereed poster).

[B6] Wilson B (2006). The challenge of developing evidence-based genetics health care in practice. Familial Cancer.

[B7] Giacomini M (2005). One of these things is not like the others: The idea of precedence in health technology assessment and coverage decisions. Milbank Quart.

[B8] Deber R (1992). Translating technology assessment into policy: Conceptual issues and tough choices. Int J Technol Assess Health Care.

[B9] Giacomini M, Miller F (2003). Confronting the "Gray Zones" of technology assessment: Evaluating genetic testing services for public insurance coverage in Canada. Int J Technol Assess Health Care.

[B10] Evans J, Skrzynia C, Burke W (2004). The complexities of predictive genetic testing. BMJ.

[B11] Health Technology Assessment Task Group on behalf of the Federal/Provincial/Territorial Advisory Committee on Information and Emerging Technologies (2004). Health technology strategy 1.0, Final report. http://www.cadth.ca/media/corporate/planning_documents/health_tech_strategy_1.0_nov2004_e.pdf.

[B12] Ontario Ministry of Health and Long Term Care (2002). Predictive genetic tests and health care costs: Final report prepared for the Ontario Ministry of Health and Long Term Care. http://www.health.gov.on.ca/english/public/pub/ministry_reports/geneticsrep02/chepa_rep.pdf.

[B13] Ontario Provincial Advisory Committee on New Predictive Genetic Technologies. Genetic Services in Ontario (2001). Mapping the future. Ontario Ministry of Health and Long Term Care. http://www.health.gov.on.ca/english/public/pub/ministry_reports/geneticsrep01/genetic_report.pdf.

[B14] Burke W (2004). Genetic testing in primary care. Ann Rev Genom Hum Genet.

[B15] Parker M, Lucassen A (2003). Concern for families and individuals in clinical genetics. J Med Ethics.

[B16] genetic medicine. (n.d.) Webster's New Millennium™ Dictionary of English, Preview Edition (v 0.9.7). http://dictionary.reference.com/browse/genetic medicine.

[B17] Patton MQ (2002). Qualitative Research and Evaluation Methods.

[B18] Bernard RH Research Methods in Anthropology: Qualitative and Quantitative Approaches.

[B19] Ginsburg ME, Kravitz RL, Sandberg WA (2000). A survey of physician attitudes and practices concerning cost-effectiveness in patient care. West J Med.

[B20] Rabino I (2007). Research scientists surveyed on ethical issues in genetic medicine: a comparison of attitudes of US and European researchers. New Genet Soc.

[B21] Joly Y (2006). Life insurers' access to genetic stalemate?. Health Law Rev.

[B22] Joly Y, Knoppers BM (2004). Physicians, genetics and life insurance. CMAJ.

[B23] Caulfield T (2004). Biotechnology and the popular press: The hype and the selling of science. Trends in Biotech.

[B24] Bubela T (2006). Science communication in transition: Genomics hype, public engagement, education and commercialization pressures. Clin Genet.

[B25] Holtzman NA (2005). The quality of media reports on discoveries related to human genetic diseases. Comm Genet.

[B26] National Health Committee, National Advisory Committee on Health and Disability (2006). District Health Board decision-making about new health interventions: A background paper. http://www.nhc.health.govt.nz/moh.nsf/pagescm/667/$File/dhb-decisions-new-health-background-paper.pdf.

[B27] Scheuner MT, Rotter JI (2006). Quantifying the health benefits of genetic tests: a clinical perspective. Genet Med.

[B28] Grosse SD, Houry MJ (2006). What is the clinical utility of genetic testing?. Genet Med.

[B29] UK Genetic Testing Network (2006). Framework for delivering the UK genetic testing network. http://www.ukgtn.nhs.uk/gtn/digitalAssets/0/277_UKGTN_Framework_March_06.pdf.

[B30] Sanderson S (2005). How can the evaluation of genetics tests be enhanced? Lessons learned from the ACCE framework and evaluating genetic tests in the United Kingdom. Genet Med.

